# Gingival metastasis of a breast carcinoma

**DOI:** 10.1002/ccr3.3216

**Published:** 2020-08-04

**Authors:** Moustapha Ndiaye, Ababacar Diegane Faye, Mame Sanou Diouf, Ciré Ndiaye, Abdou Sy, Adja Coumba Diallo, Birame Loum, Richard Deguenonvo, Evelyne Siga Diom, Malick Ndiaye, Issa Cheikh Ndiaye, Bay Karim Diallo, Abdourahmane Tall, Raymond Diouf

**Affiliations:** ^1^ Hospital Intern Dakar Senegal; ^2^ ENT Surgeon Dakar Senegal; ^3^ HOGIP Hospital Center Cheikh Anta Diop University Dakar Senegal; ^4^ FANN Hospital Center Cheikh Anta Diop University Dakar Senegal; ^5^ HED Hospital Center Thies University Thies Senegal; ^6^ Institut Joliot Curie de l'Hôpital Aristide Le Dantec Dakar Senegal; ^7^ HEAR Hospital Center Cheikh Anta Diop University Dakar Senegal; ^8^ De la Paix Hospital Ziguinchor University Ziguinchor Senegal

**Keywords:** ear, emergency medicine, nose throat, oncology

## Abstract

We report a case of metastasis to the gingiva of breast carcinoma. The breast tumor was treated with neoadjuvant chemotherapy followed by a radical mastectomy associated with axillary dissection. The gingival lesion occurred 7 months after surgery; the metastasis was confirmed by a biopsy.

## Graphical abstract

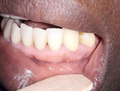

## 1 INTRODUCTION

Metastases of the oral cavity are rare, accounting for approximately 1% of all tumors of the oral cavity.1 We report a case of gingival metastasis from ductal carcinoma of the breast: metastasis occurring 7 months after surgical treatment of breast cancer despite healthy margins of resection.

This case is not an obvious diagnosis. We therefore recommend a biopsy for any chronic gingival mass in the context of previous malignancy.

## 2 CLINICAL CASE

This is a case of 43‐year‐old female patient who presented 2 years ago with ductal carcinoma of the right breast. Surgical exeresis (mastectomy + axillary lymph node dissection) had been preceded by neoadjuvant chemotherapy. The histological type was an infiltrating ductal carcinoma classified as SBR II (2+3+1) without vascular emboli or perineural invasion. Surgical resection margins were tumor‐free. Postoperative radiotherapy was not considered necessary.

Seven months after treatment of breast cancer, the patient had painless, nonhemorrhagic gingival swelling.

**FIGURE 1:**
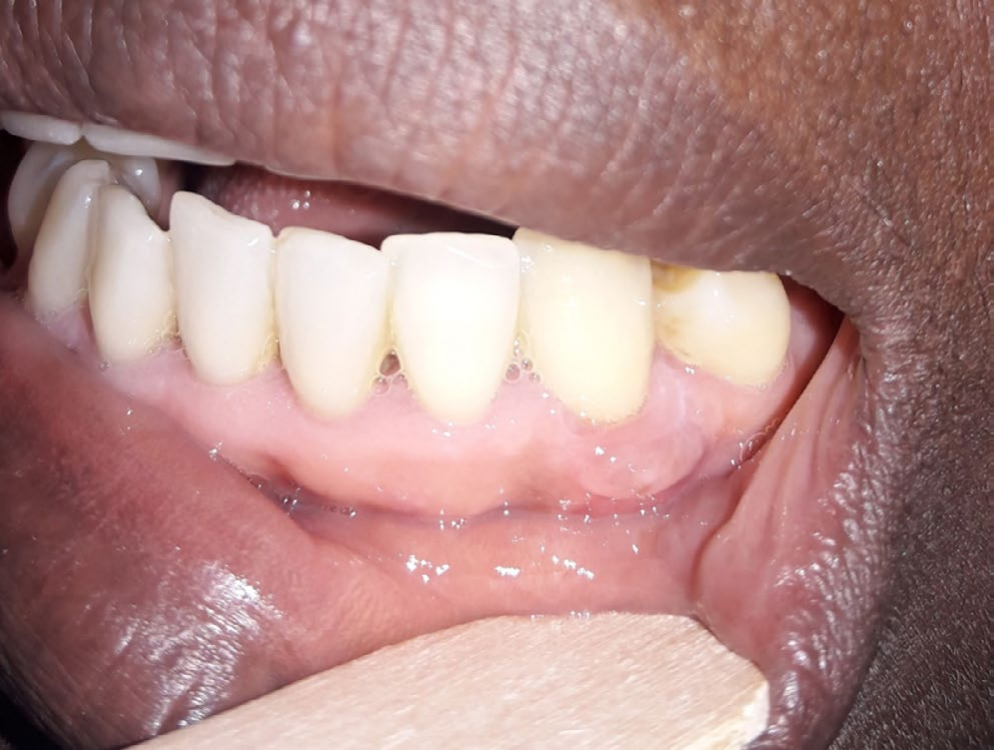
Well‐limited gum mass

On examination, there was a rounded, nonbleeding mass of lower left gingival seat near tooth 33 Figure 1. The mass was 1 cm long, and the mucosa covering it was normal in appearance.

**FIGURE 2:**
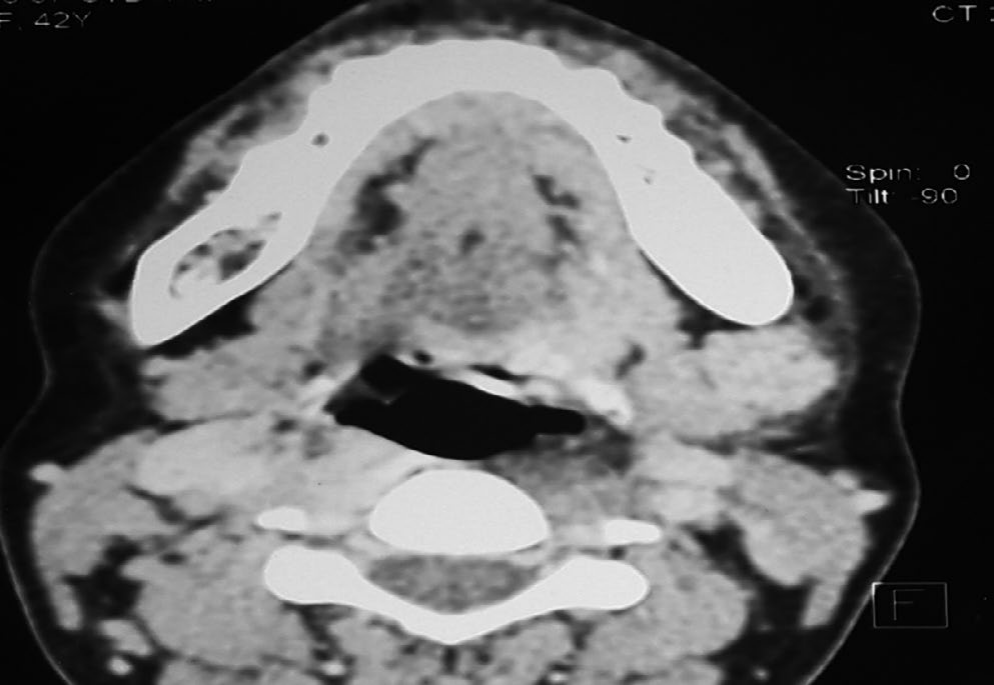
Small gingival thickening without associated bone involvement

The CT scan performed showed a small gingival thickening without bone involvement Figure 2.

A biopsy of the gingival lesion had been performed, and the histological results indicated metastasis of an infiltrating ductal carcinoma. PET scan was not available.

The lesion was excised with the underlying mandibular segment and teeth 31, 32, and 33. Treatment in addition to surgery was adjuvant chemotherapy.

No tumor markers for breast cancer had been investigated. No secondary locations were detected on whole‐body CT.

After 1 year of follow‐up, the patient had no recurrence or other metastases.

## 3 DISCUSSION

Metastases of the oral cavity are rare, accounting for approximately 1% of all tumors of the oral cavity.^1^

The three main sites of the primary tumor are the bronchopulmonary system, the breast, and the kidneys.^2^

These metastases of the oral cavity involve the mandibular bone more than the soft tissue.^1^, ^3^ The breast is no exception to the rule since it is an osteophilic cancer. Invasive ductal carcinoma is the most common form of breast cancer and accounts for 50%‐70% of invasive breast cancers.^3^

The preferred bone locations are the molar and retromolar regions.^2^, ^4^

The more frequent involvement of the mandible is linked to the presence of hematopoietic bone, decreasing blood flow, thereby promoting the deposition of cancer cells.^4^ One case of gingival metastasis in a mandibular extraction cavity has been reported.^3^

The gum represents the most frequent localization of metastases sitting in the oral mucosa (54%), followed by the tongue (25%).^2^

The diagnosis of gingival metastasis is not always obvious when the lesion does not have the characteristics of malignancy. In our case, the lesion had all the characteristics of a benign lesion (limited mass, painless, healthy‐looking skin). This lesion could be confused with an epulis or a granuloma.^2^ This means that it will be necessary to have an easy biopsy in front of any gum mass in the context of previous malignancy.

Before any treatment, a PET scan as well as the search for tumor markers must be performed. However, in our context (low‐income country), these assessments are often lacking.

Treatment, whether palliative or curative, must be discussed at a multidisciplinary concertation meeting. Surgery is performed when practicable. Neoadjuvant or adjuvant chemotherapy (based on Taxotere and doxorubicin) and flash radiotherapy are also therapeutic means.^2^, ^4^

The prognosis for metastatic lesions of the oral cavity is poor, the survival rate of mucosal lesions being less good than that of bone lesions.^2^ Maschino^2^ reports a rate of 17.4% at 4 years, while Rocha^5^ estimates survival at 10% at 4 years.

## 4 CONCLUSION

Based on the clinical characteristics of the gum mass, the diagnosis of this metastasis is not always obvious. We therefore recommend performing a biopsy on any gingival lesion in the context of previous malignancy in order to avoid diagnostic errors.

## CONFLICT OF INTEREST

None declared.

## AUTHOR CONTRIBUTIONS

MN: wrote the manuscript, and all the other authors: involved in patient care since admission to its operation and read and approved the final version of the manuscript.
